# *Sarcina ventriculi* in blood: the first documented report since 1872

**DOI:** 10.1186/1471-2334-13-169

**Published:** 2013-04-08

**Authors:** Tamara Tuuminen, Päivi Suomala, Sakari Vuorinen

**Affiliations:** 1 Haartman Institute, University of Helsinki, Department of Bacteriology and Immunology, PO Box 21, Helsinki, 00014, Finland; 2 Eastern Finland Laboratory Centre Joint Authority Enterprise (ISLAB), Mikkeli District Laboratory, Porrassalmenkatu 35-37, Mikkeli, 50100, Finland; 3 Mikkeli Central hospital, Porrassalmenkatu 35-37, Mikkeli, 50100, Finland

**Keywords:** Sarcina ventriculi, Bacteremia, Blood stream infection

## Abstract

**Background:**

In 1872, in British Medical Journal (BMJ) Dr. David Ferrier published that *Sarcina ventriculi* (Goodsir) constantly occurred in the blood of man and the lower animals. His observation was based on bleeding experiments, incubation of blood at 100^o^F (37.8^o^C) and later examination. He found “immense numbers of beautifully formed sarcinæ”. In the next issue of BMJ Dr. Charlton Bastian expressed concerns that *Sarcina* might indeed be “really a living thing” or “might be partly organic and partly mineral in its constitutions”.

**Case presentation:**

Anaerobic gram-positive giant coccae assembled in tetrads were recovered from one anaerobic blood culture bottle of a 48-year-old female who in her early childhood was diagnosed with congenital chloride diarrhoea. This is a rare recessively inherited disease that belongs to the Finnish disease heritage. The bacteria were identified with the 16S rRNA gene sequencing.

**Conclusions:**

Here, after more than a century we present the first report that *Sarcina ventriculi* can indeed cause bacteremia in a susceptible person.

## Background

In 1872, a physiologist, Dr David Ferrier at the King’s college of London published a paper in the BMJ entitled “The constant occurrence of *Sarcina ventriculi* (Goodsir) in the blood of man and the lower animals: with remarks on the nature of sarcinous vomiting” [1]. Dr. Ferrier explained that he collected blood with sufficient aseptic precautions from the carotid or other big vessels of rabbits, cats, dogs, and frogs into sterilized tubes which were then sealed into flasks which were stopped with cotton wool. Human blood was collected from a slight incision in the arm or hand. The blood was incubated at 100°F (37.8°C) and examined after 7-10 days, or in some cases after two months. He found “immense numbers of beautifully formed sarcinæ”. He wrote that the blood of frogs and humans, as well as the blood of patients in the peak of enteric fever, produced particles of the size and characteristics of *Sarcina ventriculi*, and that no other organisms of putrefactive changes were observed. He concluded that his aseptic precautions “would of itself sufficiently establish the fact of freedom from external contamination”. This communication was commented in the next issue by a pathologist, Dr. H Charlton Bastian, in his article entitled “On the nature of the co-called *Sarcina ventriculi*” [2]. He expressed concerns that Dr. Ferrier’s observations “might be partly organic and partly mineral in its constitutions” and questioned the very nature of *Sarcina* of being “really a living thing”. At that time it was assumed that “sarcinæ could be an unusual form of some one of our common mucors”. It was also believed that “the blood…is not the only source of *Sarcina*” but they may reside also in the stomach and may be associated with sarcinous vomiting in patients with gastric cancer. In his publication, Dr. Bastian referred to the finding that *Sarcinae* were also found in stagnant water and soil.

**Figure 1:**
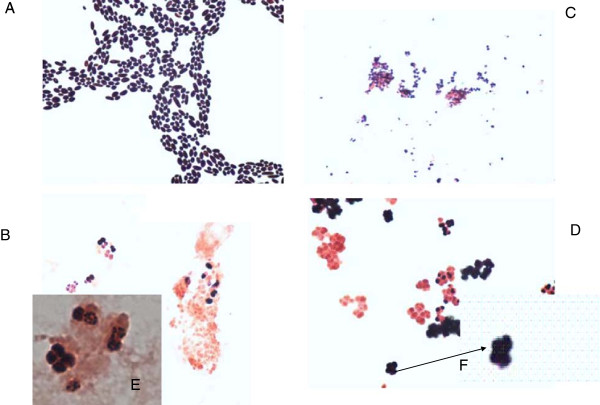
**Gram stains of four clinical isolates to illustrate the giant size and peculiar architecture of the blood isolate of *Sarcina ventriculi *(B).** To demonstrate the size of the *Sarcina* cells other images (A, C, D) are presented for comparison at the same magnification x 100. Note that the size of *Sarcinae* is as big as that of yeast and much bigger than that of *Finegoldia magna*. **A.***Candida parapsilosis,* staining from the colony. **B.***Sarcina ventriculi,* staining from an anaerobic blood culture bottle, 2 images (isolated from the patient presented). The identification was based on 16S rRNA sequencing. **C.***Finegoldia magna,* big anaerobic coccus, staining from the colony*.***D.***Sarcina*-like bacteria recovered from faeces of another patient with norovirus gastroenteritis, staining from the colony. The suspicion of *Sarcinae* was based on typical colony morphology and gram-staining. **E.***Sarcina ventriculi*, staining from an anaerobic blood culture bottle (as picture B), one image taken with magnification 1000, oil objective. Note tetrad assembling. **F.** Zoomed image of the tetrad of *Sarcina*-like bacteria (from picture D).

Later, using 16S rRNA gene sequencing *Sarcina ventriculi* was phylogenetically placed into Clostridiaceae family, Firmicutes phylum. At present 67 species of *Sarcinae* are known. These are strictly anaerobic, gram-positive giant cocci of cuboidal cell arrangements (Figure 1). They produce small, non-fluorescent, dry, greying colonies with a flower-like structure on anaerobe basal agar with 10% horse blood (ABA, Oxoid, Basingstoke, UK). In our laboratory *Sarcinae* or Sarcina-like bacteria were also recovered from faeces of a person with norovirus gastroenteritis in whom faeces was examined for *Clostridium difficile.* The bacteria grew in pure colonies after the sample was treated with ethyl alcohol, the routine procedure used for the cultivation of *Clostridium difficile*.

Below, we present a case of isolation of *Sarcina ventriculi* from the blood of a patient suffering from congenital chloride diarrhoea (CLD). This condition is a rare recessively inherited disease that belongs to the Finnish disease heritage [3]. It is caused by mutations in the *SLC26A3* gene on the chromosome 7 leading to the disturbances in the function of the major anion exchanger on the surface of intestinal epithelial cells. Untreated, the chloride-bicarbonate transport system disturbance leads to chloride, sodium and water deficiencies and chronic diarrhoea during the foetal life.

## Case presentation

A 48-year-old female was diagnosed with CLD in her early childhood. She was on a lifelong oral substitution of sodium and potassium chloride and otherwise healthy. Suddenly one evening she felt feverish, had stomach cramps, vomited 10 times and experienced extensive watery diarrhoea. She was tachycardic, but not hypotensive. She had no respiratory symptoms, and her oxygen saturation was normal. She was transferred to the ward, where her liquid and salt homeostasis was balanced*.* She had no prior antibiotic treatment, nor received any toxic substances, nor had a history of travel abroad. She was not an intravenous drug abuser. None of her family members had similar symptoms. Her C-reactive protein increased slightly from normal to 51 mg/l, and she had leucocytosis 11,2-22,6 × 10E9/l. Her secondary hypopotassemia (2,8 mmol/l) and lactic acidosis (3,40 mmol/l) were corrected, and plasma creatinine and alanine aminotransferase values returned to normal (from 126 to 72 micromol/l and from 592 to 17 U/l, respectively). Serum alkaline phosphates, γ-glutamyltransferase and bilirubin were normal. At admission she was hyperglycemic, but not during the follow-up. The abdominal ultrasound examination and thyroidal functions were normal. Faecal pathogens and *Clostridium difficile* were not recovered. Tests for viral enteritis or specific hepatitis pathogens were not done because her clinical presentation was septicaemia, and because Hepatitis A is not endemic in this region. From one anaerobic blood culture bottle gram-positive anaerobic coccae were recovered. The isolate was identified in a reference laboratory (Huslab, Helsinki, Finland) by sequencing a 528bp fragment of 16S rDNA gene as described [4]. The primers for amplification were (5'->3') AGAGTTTGATCMTGGCTCAG (position 8-27) and GTATTACCGCGGCTGCTG (position 536-519). The sequence had 100% similarity with *Sarcina ventriculi* strains with accession numbers AM902707.1; AF110272.1; NR_026146.1; D14151.1 when compared to NCBI data base using BLAST analysis.

The patient was treated with oral amoxicillin for 5 days. Since this episode she has been asymptomatic. In the absence of invasive sources (e.g. catheterisation, dialysis, and intravenous drug abuse) it seems reasonable to consider that the gastrointestinal tract may have been the source of entry of this organism into the blood stream. The patient’s symptoms were caused by *Sarcinae* blood-borne infection because she responded quickly to antimicrobial therapy.

The information on *Sarcinae* is very scarce in veterinary and human clinical microbiology textbooks with the exception of a few reports [5-8]. Colonisation of *Sarcinae* in human intestine and the influence of diet on the colonization human intestines by *Sarcinae* was investigated, and it was found that viable *Sarcinae* were detected in more than 50% of vegetarians, whereas the bacteria were not found in those with mixed diets [5]. Recently, an interesting report on five cases with *Sarcina*-like organisms identified in the upper gastrointestinal endoscopic biopsies was presented [8]. This report raised an intriguing question whether *Sarcinae* can cause disease in humans or whether it is a bystander with the stomach as their natural habitat. The authors [8] noted that *Sarcinae* have a peculiar tetrad packeting architecture. *Micrococcus* species can also occur in tetrads, however these species can be easily distinguished from *Sarcinae: Micrococcus* is regular size coccae and aerobic while *Sarcinae* are strict anaerobic, and the size of the cell is as big as of yeasts.

## Conclusions

Dr David Ferrier ended his article “Sarcinæ still remain as mysterious as ever. What is their true nature? Are they parasites, or are they a normal constituent of the blood? are question on which one might speculate, but which I reserve rather for experimental solution”. Since that time molecular biology techniques facilitating exact taxonomy of microorganisms have been developed, but the clinical significance of *Sarcinae* both in humans and animals still remains obscure. We also do not know whether we name *Sarcina* same microorganisms as our colleagues a decade ago. However, as far as we are aware this is the first report substantiated by molecular methods to document *Sarcina ventriculi* bacteremia. Interestingly, this report of *Sarcina ventriculi* blood stream infection was detected in a patient with a predisposing intestinal malfunction typical for CLD. In our country, the CLD trait is more prevalent among the population of Eastern Finland (our patient). The risk for intestinal inflammatory diseases is higher in the patients with CLD, being on the average of 6% of affected cases [9]. CLD might predispose to mucosal integrity break down leading to occasional leak of intestinal microbiota such as *Sarcina.* Exact mechanisms triggering gut inflammation in CLD patients are not yet fully elucidated. A plausible scenario is that higher chloride concentration in the lumen lowers pH of the intestinal content exposing mucosa to occasional damage. This case report emphasizes that bacteremia can be a serious sequela of *Sarcina*, in addition to the previously reported emphysematous gastritis and sarcinous vomiting. This case highlights the importance of recognizing the clinical presentation (intractable sarcinous vomiting) for a potentially serious and deadly *Sarcina* infection. In addition, given the severity of disease, recent resurgence of *Sarcina* seen in the human gut [8], and the gut being a known access point for hematologic invasion, increased recognition of this entity is important.

## Consent

Written informed consent was obtained from the patient for publication of this Case report. A copy of the written consent is available for review by the Editor of this journal.

## Competing interests

The authors declare that they have no conflict of interest.

## Authors’ contributions

TT investigated blood cultures of the patient, performed the literature survey and drafted the manuscript; PS participated in the microbiological workup; SV treated the patient and helped to draft the manuscript. All authors read and approved the final manuscript.

## Pre-publication history

The pre-publication history for this paper can be accessed here:

http://www.biomedcentral.com/1471-2334/13/169/prepub
